# A hybrid CNN-transformer model with adaptive activation function for potato leaf disease classification

**DOI:** 10.1038/s41598-025-34406-4

**Published:** 2026-01-06

**Authors:** Ayan Mondal, Ayan Chatterjee, Nurilla Avazov

**Affiliations:** 1 School of Electrical Engineering, Aalto University, Otakaari 1B, 02150 Espoo, Finland; 2 Department of Digital Technology, NILU, Instituttveien 18, 2007 Kjeller, Norway; 3 Inland School of Business and Social Sciences, University of Inland Norway, Vormstuguvegen 2, 2624 Lillehammer, Norway

**Keywords:** Activation function, AFpM, Classification, CNN, PFpM, Potato plant disease, PLDNet, Computational biology and bioinformatics, Engineering, Mathematics and computing, Plant sciences

## Abstract

Potato plants are highly vulnerable to numerous diseases that can substantially affect both yield and quality. Conventional approaches for detecting these diseases are often labor-intensive, slow, and prone to inaccuracies, particularly under variable environmental conditions. This study presents a hybrid deep learning architecture, termed potato leaf diseases DenseNet **(PLDNet)**, which integrates a DenseNet-based convolutional neural network with a Transformer-based attention module to accurately classify potato leaf diseases. Furthermore, an adaptive parametric activation function, referred to as **Adaptive Flatten p-Mish (AFpM)**, is proposed to enhance the model’s learning flexibility and representational capacity. When evaluated on the PlantVillage and Mendeley datasets, PLDNet attains classification accuracies of 99.54% and 87.50%, respectively, surpassing contemporary state-of-the-art models and activation techniques. The proposed framework exhibits strong generalization performance and offers a scalable, efficient approach for automated plant disease identification. To highlight the novelty, the proposed AFpM activation function introduces a learnable parameter enabling adaptive nonlinearity, improving over Mish, Swish, and PFpM activation functions through dynamic gradient control. AFpM improves accuracy by 2.52% on Mendeley dataset, and 1.93% on PlantVillage dataset compared to PFpM, and by more than 3% compared to Swish and Mish.

## Introduction

Agricultural productivity remains vital for ensuring global food security, sustaining economic development, and promoting rural prosperity. With the continuous rise in population and the growing impact of climate change, maintaining plant health has become increasingly essential^1,2^. Contemporary technologies, particularly machine learning and deep learning, are being progressively utilized in agriculture to enhance disease identification and improve crop productivity^1–3^. These technological innovations hold significant promise for smallholder farmers in developing nations who often lack access to expert agronomic support. Agriculture continues to serve as a cornerstone of human civilization, particularly within developing economies, where it underpins both national income and the livelihoods of large portions of the population^4^. Nonetheless, the sector faces persistent constraints such as climatic variability, soil degradation, and the proliferation of plant pathogens^5^. Among major food crops, the potato stands out as one of the most economically important, ranking fourth in global consumption^6^. This crop is highly susceptible to numerous diseases caused by fungi, bacteria, nematodes, and viruses, all of which can drastically reduce yield and quality^7^. Hence, timely and accurate disease detection is essential for effective management practices. Traditional diagnostic techniques, however, often rely on manual observation or laboratory-based testing, which are laborious, time-intensive, and prone to human error^8,9^. Furthermore, the visual resemblance of disease symptoms, such as between early and late blight-renders precise identification difficult without specialized expertise or molecular tools^10^. In addition, variations in environmental parameters including humidity, temperature, and soil composition can alter disease expression and hinder prompt diagnosis^11,12^.

To address these challenges, machine learning techniques have evolved as reliable and efficient tools for identifying plant diseases. In recent years, several researchers have focused on developing automated systems for the detection of potato leaf infections^13–31^. Early studies primarily depended on handcrafted feature extraction and conventional machine learning classifiers^13,14^, which often made the feature design process laborious and sensitive to variations in image quality. To overcome these limitations, Convolutional Neural Networks (CNNs) have shown outstanding capabilities in automatically learning discriminative features and enhancing classification accuracy across a range of image recognition applications, including plant leaf disease categorization^15–31^. Nevertheless, these models still encounter difficulties in achieving consistent performance when trained on complex, real-world leaf images and maintaining high classification accuracy under varying environmental conditions.

Motivated by recent advancements in deep learning^32–39^, particularly in 2-dimensional Convolutional Neural Networks (2-D CNNs), we propose an automatic potato leaf disease image classification framework named **PLDNet**. In addition, we introduce a new activation function for deep learning, termed **Adaptive Flatten p-Mish (AFpM)**, which enhances the adaptability and precision of disease prediction from image data. To assess the effectiveness of the proposed approach, two widely recognized and distinct potato leaf datasets were employed − the PlantVillage dataset^40^, containing images captured under controlled conditions, and the Mendeley dataset^28^, featuring images with diverse and complex natural backgrounds. Experimental evaluations demonstrate that the proposed **PLDNet** model achieved an overall accuracy of 99.54% on the PlantVillage dataset and 87.50% on the Mendeley dataset, surpassing the performance of existing state-of-the-art architectures. Unlike existing CNN or Transformer-based approaches, the novelty of the proposed work lies in (a.) a hybrid DenseNet–Transformer architecture designed specifically to address the real-world potato disease image variability, and (b.) the introduction of a learnable activation function (AFpM) that adapts its nonlinear behavior during model training. No prior work has jointly explored hybrid feature extraction and adaptive activations for potato leaf disease classification. The primary contributions of this study are summarized as follows:

- Development of a CNN-based architecture, **PLDNet**, optimized for automated potato leaf disease classification.
- Design of a novel activation function, **Adaptive Flatten p-Mish (AFpM)**, aimed at enhancing CNN learning efficiency in challenging visual contexts.
- Comprehensive benchmarking of the proposed model on two benchmark datasets − PlantVillage (controlled setting) and Mendeley (uncontrolled setting) − demonstrating superior classification accuracy.
- Comparative evaluation with established models to validate the robustness and generalization capability of **PLDNet** across varying environmental conditions.
- Presentation of a reproducible and extensible framework applicable to disease identification in other crop species.

The remainder of this paper is organized as follows. Section 2 provides a detailed review of related literature. Section 3 outlines the proposed methodology. Section 4 reports and discusses the experimental outcomes along with potential directions for improvement. Finally, Section 5 concludes the study.

## Related work

Over the last decade, significant progress has been observed in the field of automated plant disease detection, particularly through the use of leaf imagery. This advancement has been primarily facilitated by deep learning techniques and the availability of extensive benchmark datasets such as PlantVillage^40^ and Mendeley^28^. Traditional machine learning algorithms, including Support Vector Machines (SVMs) and Random Forests, initially established the foundation for this research area by illustrating that handcrafted image descriptors could deliver reliable classification results. However, with the emergence of deep learning, Convolutional Neural Networks (CNNs) have revolutionized the domain by learning discriminative hierarchical features directly from image data, thereby surpassing the performance of conventional methods.

Numerous investigations have reported high classification accuracies on well-controlled datasets such as PlantVillage, employing architectures that range from basic CNNs to advanced variants like ResNet, DenseNet, EfficientNet, and hybrid ensemble frameworks. Despite these improvements, the generalization capability of such models remains a challenge, as evident from their reduced performance on complex and realistic datasets such as Mendeley. Moreover, the majority of studies emphasize architectural optimization and ensemble learning, while the influence of activation functions from fundamental to non-linearity and model convergence has received comparatively little attention. This underrepresentation constitutes a notable research gap, as prevailing models predominantly employ standard activation functions such as ReLU or Mish without systematically assessing their behavior in noisy or imbalanced agricultural image conditions.

To bridge this gap, the present study proposes a novel activation function (AFpM) and a CNN-based model (PLDNet) to improve robustness and generalization across both controlled and natural environments. The following sections provide a comprehensive analysis of recent works, emphasizing datasets, model architectures, performance outcomes, and emerging research trends. Initial efforts in potato leaf disease recognition primarily relied on conventional machine learning algorithms, which achieved commendable performance but suffered from limited scalability. For instance, Islam et al. (2017)^13^ implemented a multiclass Support Vector Machine (SVM), obtaining an accuracy of 95% on the PlantVillage dataset. Similarly, Iqbal et al. (2020)^14^ combined global image descriptors with a Random Forest classifier and achieved 97% accuracy. These findings illustrate that well-crafted feature extraction pipelines enable traditional approaches to achieve competitive results in structured image datasets.

### Traditional machine learning approaches

Initial efforts in potato leaf disease recognition primarily relied on conventional machine learning algorithms, which achieved commendable performance but suffered from limited scalability. For instance, Islam et al. (2017)^13^ implemented a multiclass Support Vector Machine (SVM), obtaining an accuracy of 95% on the PlantVillage dataset. Similarly, Iqbal et al. (2020)^14^ combined global image descriptors with a Random Forest classifier and achieved 97% accuracy. These findings illustrate that well-crafted feature extraction pipelines enabled traditional approaches to achieve competitive results in structured image datasets.

### Deep learning and CNN-based approaches

With the growing prominence of deep learning, neural network-based approaches have become the leading choice for automated feature extraction and classification. Tiwari et al. (2020)^15^ integrated VGG19 as a feature extractor with logistic regression, reporting an accuracy of 97.8% on the PlantVillage dataset. Kumar et al. (2021)^16^ adopted a Feedforward Neural Network (FFNN), achieving 96.5% accuracy on the same dataset. Tambe et al. (2023)^19^ attained a top accuracy of 99.18% using a conventional CNN model, whereas Lanjewar et al. (2024)^27^ enhanced the performance to 99% by employing a modified DenseNet structure. Additional CNN-based studies conducted by Patil et al. (2023)^18^, Kumar et al. (2023)^22^, Khan et al. (2024)^25^, and Roy et al. (2024)^24^ achieved accuracies between 97.16% and 98.83%, reaffirming the effectiveness of CNN architectures in well-curated datasets such as PlantVillage.

### Advanced architectures and hybrid models

Deeper and optimized networks have also demonstrated strong performance in potato leaf disease recognition. Paul et al. (2024)^26^ implemented ResNet-50, achieving 98.79% overall accuracy, while Muthuraja et al. (2023)^20^ applied ResNet-152 and obtained 98.78%. Similarly, Ali et al. (2022)^17^ utilized an Enhanced EfficientNet model that reached 97.22%, highlighting that deeper and fine-tuned architectures yield improved generalization. Mathur et al. (2024)^23^ compared multiple CNN variants and found that VGG19 delivered the best performance at 93%, suggesting performance limitations in earlier CNN designs. Furthermore, hybrid and ensemble networks have contributed to additional accuracy gains. For example, Arshad et al. (2023)^21^ combined VGG19 and Inception-V3, achieving 98.66%, while Shabrina et al. (2023)^28^ employed EfficientNetV2B3 to reach 98.15% on the PlantVillage dataset. These findings collectively emphasize that model fusion and architectural integration can enhance generalization across different visual domains.

In contrast, results obtained from the real-world Mendeley dataset exhibit a noticeable reduction in accuracy due to uncontrolled imaging conditions and environmental variability. Shabrina et al. (2023)^28^ observed an accuracy of 73.63% using EfficientNetV2B3 on Mendeley, compared to 98.15% on PlantVillage with the same model. Sinamenye et al. (2025)^29^ improved upon this by incorporating a hybrid EfficientNetV2B3–Vision Transformer (ViT) architecture, achieving 85.06%. Similarly, Mhala et al. (2025)^30^ and Park et al. (2025)^31^ reported 81.31% and 78.14% accuracy using DenseNet201 and RCA-Net, respectively. Despite notable architectural differences, Densenet models tend to rely on dense feature reuse, ResNet variants often struggle with shallow texture cues, EfficientNet’s compound scaling does not generalise well beyond clean datasets, and recent ViT-based hybrids require large and high-quality training samples, resulting in only modest performance gains on controlled datasets and exposing their limited ability to handle real-world variability. These outcomes indicate that while CNN-based and hybrid models dominate in structured scenarios, their robustness declines under non-uniform conditions such as those represented in Mendeley. Therefore, developing architectures capable of adapting to diverse real-world environments remains a critical direction for future work.

### Applications of existing models, research gaps and limitations

The reviewed models have been widely applied across various agricultural contexts. One of the primary applications lies in early disease diagnosis within precision agriculture, where timely identification of infected plants contributes to improved crop yield and sustainable farming practices. Additionally, these models have been deployed in mobile and unmanned aerial vehicle (UAV)-based systems, enabling real-time field monitoring and rapid detection of disease symptoms across large cultivation areas. Beyond detection, deep learning frameworks such as CNNs have also been integrated into smart farming platforms that support automated disease alerting and decision-making processes, thereby enhancing the overall efficiency of agricultural management and reducing manual inspection efforts. Collectively, these applications highlight the transformative role of AI-driven visual recognition systems in modern agriculture.

CNN-based frameworks, such as ResNet, DenseNet, and EfficientNet, have shown particular utility in embedded systems due to their balance between accuracy and computational efficiency. Hybrid approaches further extend these applications to scenarios demanding adaptability under natural lighting and occlusion conditions. A review of existing studies reveals several persistent gaps in automatic potato leaf disease classification. Existing models have primarily achieved strong classification performance in controlled datasets, such as PlantVillage, where lighting and background conditions are uniform. However, they often fail on datasets like the Mendeley dataset, where the images are captured under uncontrolled imaging conditions due to background clutter, uneven lighting, symptom similarity, and class imbalance. These challenges remain inadequately addressed in prior literature, which motivates the necessity for a more adaptive feature extraction mechanism. Consequently, a key shortcoming is the limited investigation into activation functions specifically tailored for plant disease imagery. Although numerous architectures and optimization strategies have been tested, most research continues to rely on conventional functions like ReLU^41^ without assessing their domain suitability. The advantages of alternative activations, such as mitigating issues like bias shift^42^ and neuron inactivity^43^, have not been extensively explored. Moreover, many high-performing ensemble and feature-fusion methods impose considerable computational costs, limiting their real-time applicability in agricultural contexts. These limitations reveal that no single family of models is sufficient for capturing all types of patterns from images. CNN models are efficient at extracting fine-grained local textures, but often fail to capture long-range relationships. Transformer-based models provide global reasoning but require large, clean datasets to remain stable and fixed-form activation functions lack adaptability under noisy real-world imaging conditions. This research lacuna motivates the integration of CNNs for localised feature enrichment, a Transformer network for global context modelling, and the AFpM activation for adaptive nonlinear representation. Together, these components tackle the domain-shift problem, lighting variability, and feature ambiguity issues.

### Novel contribution - AFpM and PLDNet

To address these challenges, the present study introduces a novel activation function termed Adaptive Flatten p-Mish (AFpM) and a customized CNN architecture called PLDNet, built upon a densely connected framework^44^. Our proposed PLDNet model combines dense local feature extraction with global contextual modelling that allows the architecture to cope with background clutter, uneven lighting, and symptom similarity. In addition, the AFpM activation introduces adaptive nonlinear behaviour that alleviates gradient saturation and instability, issues frequently observed in existing models under real-world imaging conditions. Using the PlantVillage^40^ and Mendeley^28^ datasets as evaluation benchmarks, we comprehensively assess the performance of the proposed model. The comparative analysis includes state-of-the-art deep CNN architectures such as DenseNet201^44^, DenseNet169^44^, InceptionV3^45^, ResNet50^46^, VGG16^46^, and MobileNetV2^47^. Table 1 summarizes previous research on the PlantVillage and Mendeley datasets, detailing the models used, performance metrics, and whether activation functions were investigated in each case. Table 1 summarizes previous research on the PlantVillage and Mendeley datasets, detailing the models used, performance metrics, and whether activation functions were investigated in each case.

**Table 1:** A qualitative comparison of recent studies on potato leaf disease classification.

**Author (Year)**	**Model**	**Dataset**	**Activation function explored**	**Real-world evaluation**	**Accuracy (%)**
Islam et al. (2017)^13^	SVM	PlantVillage	✗	✗	95.00
Iqbal et al. (2020)^14^	Global Feature + RF	PlantVillage	✗	✗	97.00
Tiwari et al. (2020)^15^	VGG19 + Logistic Reg.	PlantVillage	✗	✗	97.80
Kumar et al. (2021)^16^	FFNN	PlantVillage	✗	✗	96.50
Ali et al. (2022)^17^	Enhanced EfficientNet	PlantVillage	✗	✗	97.22
Patil et al. (2023)^18^	CNN	PlantVillage	✗	✗	98.00
Tambe et al. (2023)^19^	CNN	PlantVillage	✗	✗	99.18
Muthuraja et al. (2023)^20^	ResNet152	PlantVillage	✗	✗	98.78
Arshad et al. (2023)^21^	VGG19 + InceptionV3	PlantVillage	✗	✗	98.66
Kumar et al. (2023)^22^	CNN	PlantVillage	✗	✗	98.83
Mathur et al. (2023)^23^	VGG19	PlantVillage	✗	✗	93.00
Roy et al. (2024)^24^	CNN	PlantVillage	✗	✗	97.16
Khan et al. (2024)^25^	CNN	PlantVillage	✗	✗	97.33
Paul et al. (2024)^26^	ResNet-50	PlantVillage	✗	✗	98.79
Lanjewar et al. (2024)^27^	Modified DenseNet	PlantVillage	✗	✗	99.00
Shabrina et al. (2023)^28^	EfficientNetV2B3	PlantVillage Mendeley	✗	✓	98.15 73.63
Sinamenye et al. (2025)^29^	EfficientNetV2B3 + ViT	PlantVillage Mendeley	✗	✓	98.15 85.06
Mhala et al. (2025)^30^	DenseNet201	Mendeley	✗	✓	81.31
Park et al. (2025)^31^	RCA-Net	Mendeley	✗	✓	78.14
**Our study**	**PLDNet + AFpM**	**PlantVillage Mendeley**	✓	✓	**99.54 87.50**

Furthermore, the performance of the AFpM activation function was thoroughly evaluated against six well-established activation functions, including ReLU^41^, Leaky ReLU^48^, GELU^49^, Swish^50^, Mish^51^, and PFpM^52^. The experimental analysis demonstrates that the proposed PLDNet model, when combined with the AFpM activation function, attains outstanding classification performance, achieving 99.54% overall accuracy on the PlantVillage dataset^40^ and 87.50% on the Mendeley dataset^28^. These results highlight the robustness and effectiveness of the developed framework for potato leaf disease classification. These contributions aim to advance automated agricultural vision systems toward improved generalization, and real-time feasibility. The main contributions of this study are summarized as follows:

- A detailed comparative evaluation of the PLDNet model incorporating the AFpM activation function with several state-of-the-art architectures,
- A systematic comparison of the AFpM activation function with other contemporary activation functions, considering PLDNet as the baseline network, and
- Provision of the PLDNet implementation on GitHub for public access upon acceptance of the manuscript.

## Methods

This section elucidates the proposed methodology adopted for the automatic classification of potato leaf diseases. We have designed a novel activation function named AFpM and proposed a hybrid CNN-Transformer model, PLDNet. We have also implemented appropriate pre-processing and data augmentation techniques to enhance classification accuracy, as discussed in the following subsections.

### Dataset description

In this study, two well-established datasets were employed to assess the effectiveness of the proposed approach for potato leaf disease classification. The first dataset utilized is the PlantVillage dataset^40^, which comprises 2,152 images divided into three distinct categories, such as *Early Blight*, *Late Blight*, and *Healthy*. Among them, 1,000 images represent diseased leaves, while 152 correspond to healthy samples. The *Early Blight* class represents potato leaves affected by the fungal pathogen *Alternaria Solani*, typically characterized by concentric brown lesions and chlorotic zones around infected areas. The *Late Blight* category includes leaves infected by *Phytophthora Infestans*, which manifests as irregular dark patches and rapid tissue decay under humid conditions. The *Healthy* class consists of disease-free potato leaves exhibiting uniform green coloration and intact surface morphology. The PlantVillage dataset^40^ was acquired under controlled laboratory conditions at an experimental farm at Penn State University, USA. All images were captured using DSLR cameras under consistent illumination, uniform backgrounds, and minimal environmental interference. Each image is stored in RGB format with a spatial resolution of 256 $\times$ 256 pixels.

**Fig. 1:**
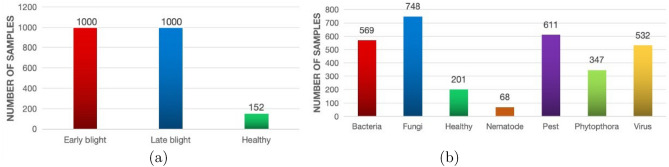
Class-wise data size: (**a**) The PlantVillage dataset; (**b**) The Mendeley dataset.

**Fig. 2:**
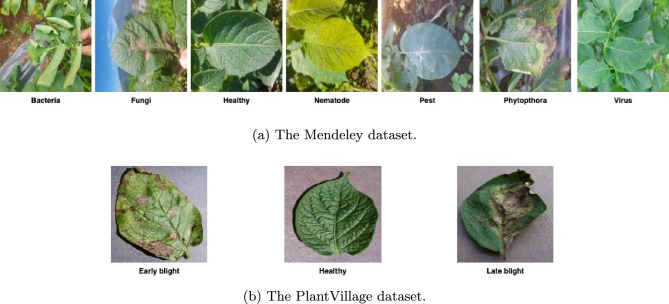
Sample images from each class for both datasets. (**a**) The Mendeley dataset; (**b**) The PlantVillage dataset.

The second dataset, referred to as the Potato Leaf Disease dataset^28^, is a recently published collection gathered under natural field conditions. This open-access dataset, hosted on Mendeley^28^, consists of 3,076 images distributed across seven categories: healthy, virus, phytophthora, nematode, fungi, bacteria, and pest. The images were captured from multiple potato farms located in Magelang and Wonosobo, Central Java, Indonesia, using various smartphone cameras in uncontrolled environments. All images are provided in JPG format with a resolution of 1500 $\times$ 1500 pixels. The class distribution of both datasets is illustrated in Figure 1, where the X-axis denotes the class labels and the Y-axis represents the corresponding image counts. The Mendeley dataset exhibits a notable data imbalance. The classes such as virus, phytophthora, nematode, fungi, bacteria, pest, and healthy contain 532, 347, 68, 748, 569, 611, and 201 images, respectively. Figure 2 presents representative samples from each class in both datasets, highlighting the diversity of image types across the prediction categories. From Figure 2, it is evident that the Mendeley dataset includes more significant real-world image variations, such as uneven lighting, partial occlusion of leaves, shadows, and complex backgrounds. These factors introduce substantial noise into the data, making the Mendeley dataset considerably more challenging and providing a realistic benchmark for evaluating model robustness.

### Data pre-processsing

As previously mentioned, the images in the PlantVillage dataset^40^ have a resolution of 256$\times$256 pixels, whereas those in the more recently introduced Mendeley dataset^28^ possess a resolution of 1500$\times$1500 pixels. For consistency in model input dimensions, all images from both datasets were resized to 224$\times$224 pixels. Each image was normalized to a pixel intensity range between 0 and 1 using min–max normalization. The datasets were then divided into training and testing subsets following an 80:20 stratified split, ensuring proportional representation of each class across both subsets. Additionally, to improve model robustness and generalization, several data augmentation strategies were applied to the training data. These included random zooming within a factor of 0.2–0.3, rotations between $-40^{\circ }$ and $40^{\circ }$, and both vertical and horizontal flipping.

### Proposed activation function

The activation function^41^ plays a fundamental role in convolutional neural networks (CNNs), as it introduces non-linearity that enables the network to model complex relationships between inputs and outputs. Among various options, the Rectified Linear Unit (ReLU)^41^ remains one of the most widely adopted due to its computational efficiency and simplicity. Nevertheless, ReLU suffers from certain limitations, including the problem of inactive neurons^43^ and the bias shift phenomenon^42^. To mitigate these issues, the Leaky ReLU function^48^ was introduced, allowing a small gradient to flow even for negative inputs. Further enhancements were achieved through the Gaussian Error Linear Unit (GELU) proposed by Hendrycks et al.^49^, which improves learning behavior but lacks adaptability because of its fixed formulation. Subsequently, several non-monotonic activation functions, such as Swish^50^ and Mish^51^, were developed, offering superior classification accuracy compared to ReLU and Leaky ReLU in many deep learning tasks. Despite this, Eger et al.^53^ reported instability concerns with the Swish function. The mathematical forms of these established activation functions are presented in Table 2.

**Table 2:** Mathematical expressions of different activation functions.

Activation function	Mathematical expression
ReLU (2010)^41^	$$f(x) = {\left\{ \begin{array}{ll} x, & \text {if } x \ge 0 \\ 0, & \text {if } x < 0 \end{array}\right. }$$
Leaky ReLU (2013)^48^	$$f(x) = {\left\{ \begin{array}{ll} x, & \text {if } x \ge 0 \\ a x, & \text {if } x < 0 \quad (a = 0.01) \end{array}\right. }$$
GELU (2016)^49^	$f(x) \approx 0.5x \left( 1 + \tanh \left[ \sqrt{\frac{2}{\pi }} \left( x + 0.044715x^3\right) \right] \right)$
Swish (2017)^50^	$f(x) = \frac{x}{1 + e^{-x}}$
Mish (2019)^51^	$f(x) = x \cdot \tanh (\ln (1 + e^x))$
PFpM (2022)^52^	$$f(x) = {\left\{ \begin{array}{ll} x \cdot \tanh (\ln (1 + e^x)) - 0.2, & \text {if } x \ge 0 \\ -0.2, & \text {if } x < 0 \end{array}\right. }$$

In this study, we introduce a non-linear and parametric activation function termed Adaptive Flatten p-Mish (AFpM), developed as an improved variant of the earlier PFpM function^52^. The mathematical formulation of AFpM is presented in Equation (1), where *z* denotes the input neuron data, and *p* represents a trainable parameter associated with each neuron. The parameter *p* follows the GlorotNormal distribution^54^, i.e., $p \sim \mathcal {N}\left( 0, \sigma ^2\right)$ with mean zero and variance $\sigma ^2$ where $\sigma ^2 = \frac{2}{n_{in} + n_{out}}$ and optimized throughout the training process via backpropagation^55^. The terms $n_{in}$ and $n_{out}$ represent the number of input and output units (neurons) connected to a given layer, respectively. This initialization ensures that the variance of activations remains stable, facilitating faster convergence and improved gradient flow during training.

**Fig. 3:**
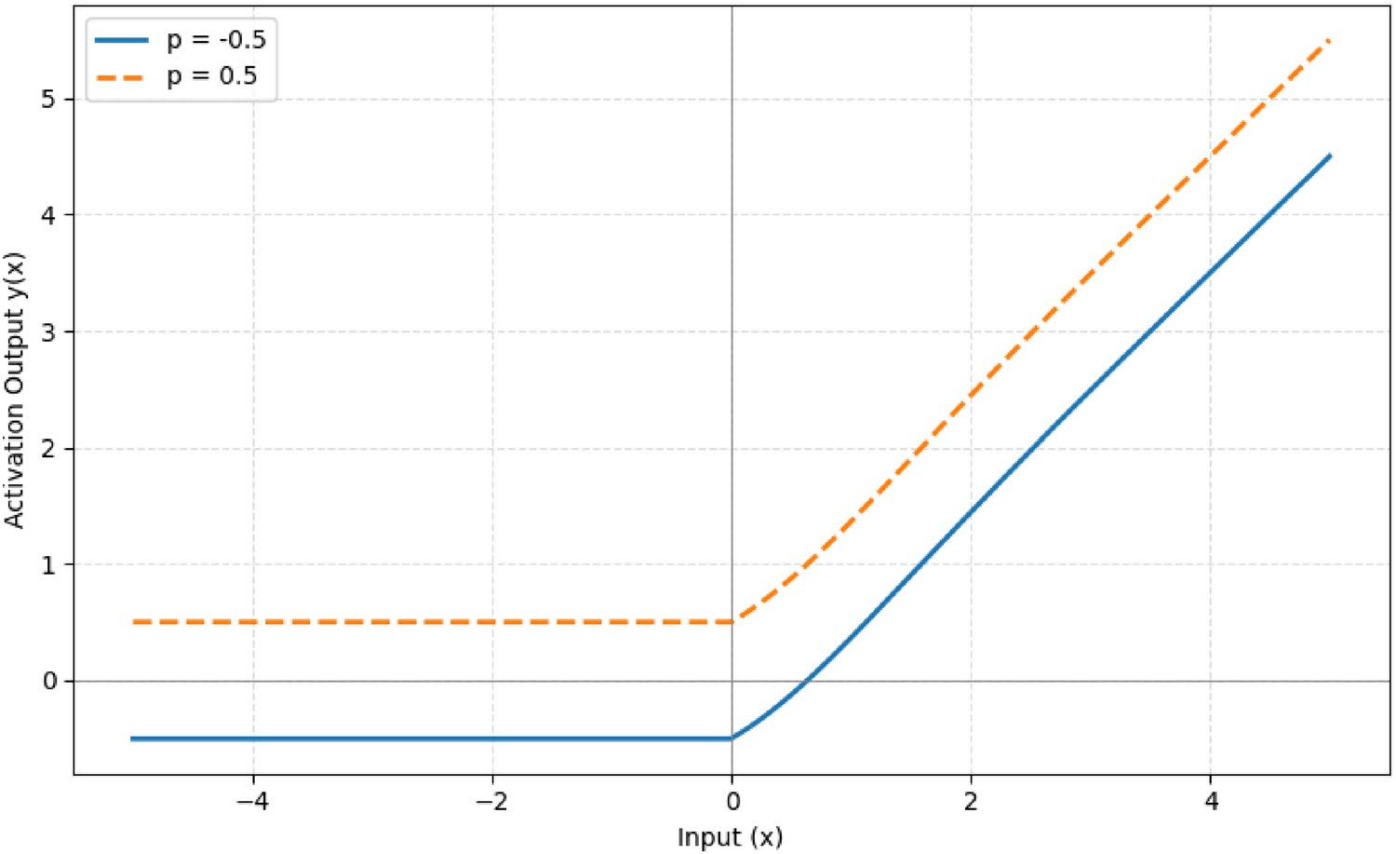
Proposed activation function, AFpM.

The subscript *j* in the equations refers to the $j^{th}$ neuron within a given layer. Equation (2) describes the optimization update rule for the parameter $p_j$ during model training, where $\lambda$ represents the learning rate, *E* is the objective (or loss) function, and $\frac{\partial y(z_j)}{\partial p_j}$ defines the sensitivity of the AFpM output with respect to $p_j$. The derivative term $\frac{\partial y(z_j)}{\partial p_j}$ is equal to 1 for all input instances, while the derivative of AFpM with respect to *z* is provided in Equation (3). A graphical representation of the proposed AFpM activation function is shown in Figure 3.

1 $$\begin{aligned} y(z) = {\left\{ \begin{array}{ll} z \cdot \tanh (\ln (1 + e^z)) + p, & \text {if } z \ge 0, \\ p, & \text {if } z < 0. \end{array}\right. } \end{aligned}$$

2 $$\begin{aligned} p_{j+1} \leftarrow p_j + \lambda \cdot \frac{\partial E}{\partial y(z_j)} \cdot \frac{\partial y(z_j)}{\partial p_j} \end{aligned}$$

3 $$\begin{aligned} \frac{dy}{dz} = {\left\{ \begin{array}{ll} \tanh (\ln (1 + e^z)) + z \cdot \text {sech}^2(\ln (1 + e^z)) \cdot \frac{e^z}{1 + e^z}, & \text {if } z \ge 0, \\ 0, & \text {if } z < 0. \end{array}\right. } \end{aligned}$$

Here, *y*(*z*) represents the output of the AFpM activation function, *z* is the pre-activation input, $p_j$ is the learnable parameter corresponding to the $j^{th}$ neuron, $\lambda$ is the learning rate controlling the update step, and *E* denotes the loss function minimized during training. The function $\tanh (\cdot )$ and $\text {sech}(\cdot )$ correspond to the hyperbolic tangent and hyperbolic secant functions, respectively.

While most existing parametric activation functions primarily aim to adjust the slopes of their functional segments, the proposed AFpM instead modifies the hinge point, denoted as *p*, thereby preserving the overall structural behavior of the function. The main difference between PFpM^52^ and AFpM lies in the treatment of the parameter *p*. In the PFpM formulation^52^, this parameter is fixed at a constant value of $-0.2$. In contrast, AFpM initializes *p* using the GlorotNormal approach and subsequently refines it through backpropagation during network training^55^. The learnable nature of *p* in AFpM enhances adaptability and flexibility, allowing the model to adjust its activation behavior according to the underlying data distribution. This parametric design enables controlled vertical shifts in the activation curve to determine an optimal *p* value for each context. As a result, individual layers of the network exhibit unique activation characteristics. This learnable parameter, *p*, is optimised jointly with network weights during training, allowing the activation shape to adapt dynamically to the underlying feature distribution. However, AFpM exhibits similar computational complexity to Mish and Swish as it employs similar fundamental operations, such as exponential, logarithm, and hyperbolic tangent, but differs only in the inclusion of a learnable scalar term. This addition does not alter the asymptotic complexity and introduces a negligible runtime overhead. Table 3 presents a qualitative comparison of AFpM with other activation functions, highlighting key distinctions that emphasize its novelty.

**Table 3:** Comparison of the proposed AFpM activation function with existing functions.

Activation function	Non-linearity	Monotonicity	Adaptivity (Trainable)	Gradient flow stability	Real-world classification performance
ReLU^41^	Moderate	Yes	No	Poor (dying neurons)	Moderate
Leaky ReLU^48^	Moderate	Yes	Fixed Slope	Improved over ReLU	Moderate
GELU^49^	High	Yes	No	Stable	Moderate
Swish^50^	High	No	No	Unstable^53^	High (PlantVillage)
Mish^51^	High	No	No	Stable	High (PlantVillage)
PFpM^52^	High	No	No (fixed $p=-0.2$)	Stable	High (synthetic)
**AFpM (Proposed)**	High	No	**Yes (trainable ** *p***)**	**Highly stable (layer-wise adaptation)**	**Superior (PlantVillage: 99.54%, Mendeley: 87.50%)**

### Proposed PLDNet model description

**Fig. 4:**
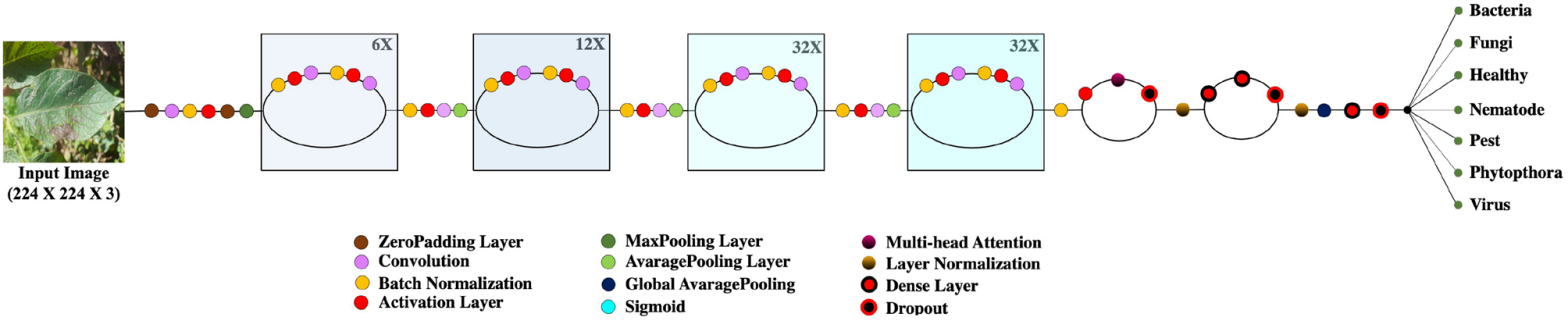
Proposed PLDNet Model architecture.

PLDNet represents a novel deep learning framework developed to address the classification task for potato leaf disease images. The model integrates the strengths of CNNs and a Transformer-based attention mechanism to effectively extract both localized visual features and long-range contextual information from leaf imagery^56^. The overall architecture of PLDNet is illustrated in Figure 4. In constructing PLDNet, the DenseNet169 network^44^, pre-trained on the ImageNet dataset, serves as the feature extraction backbone to derive detailed hierarchical representations of leaf textures and disease characteristics. DenseNet169^44^ employs densely connected layers that promote efficient feature reuse, mitigate the vanishing gradient issue, reduce parameter redundancy, and improve gradient propagation throughout the training process. The 7×7×1664 convolutional feature maps generated by DenseNet169 are then forwarded directly into a lightweight customized Transformer block^56^, enabling the attention module to operate on spatially structured feature representations without flattening. The Transformer block incorporates a single multi-head attention layer with eight heads, each attending a 208-dimensional subspace (derived from the 1664-channel input), followed by a 2048 unit feedforward module and standard residual normalization operations. This Transformer component enables the model to capture global contextual dependencies and relationships among fine-grained patterns within the leaf images. Following the Transformer block, global average pooling is applied to compress spatial dimensions and generate a compact feature vector. The final classification module includes a dense layer with 512 units, followed by an activation layer, a dropout layer (dropout rate of 0.6), and a softmax-activated output layer for multi-class classification. In total, PLDNet contains approximately 32 million trainable parameters, reflecting the combination of DenseNet backbone and the additional capacity introduced by the Transformer layer.

#### Justification of AFpM for potato leaf classification

The AFpM activation function addresses complex agricultural image classification challenges, such as high intra-class variability, noise, and uneven illumination. Potato leaf diseases often exhibit overlapping visual symptoms, such as the similarities between early and late blight, while the decision boundaries between classes are very subtle. Traditional activation functions (such as ReLU or Mish) often produce static nonlinear transformations, which limit the network’s ability to adapt to these subtle differences. AFpM introduces a learnable parameter component, $p$, which can be dynamically adjusted during training. This flexibility enables the model to tailor the activation response to better fit the data distribution, thereby improving feature recognition in challenging noisy environments, such as real-world agricultural conditions. Another advantage of AFpM lies in its smooth and non-monotonic nature, which preserves gradient flow and reduces vanishing or exploding gradients, common problems in deep networks. When integrated with PLDNet, AFpM improves convergence and enhances learning robustness, particularly when working with high-resolution leaf images from datasets such as Mendeley. Compared to standard activation functions, AFpM is able to adapt to the statistical patterns of the potato leaf disease dataset, enabling the model to focus on the complex textures and disease patterns that are critical for accurate classification. While its effectiveness has been demonstrated in this field, the proposed activation function was designed with generality in mind, enabling its extension to other agricultural and non-agricultural image classification tasks.

#### Algorithmic description of PLDNet with AFpM

The proposed Algorithm 1 integrates a DenseNet169 backbone with a Transformer-based attention block and a novel AFpM activation function to classify potato leaf diseases from RGB images. The overall time complexity of PLDNet is $O(L \cdot D^2 \cdot K^2 + H \cdot N^2 \cdot d)$, where $L$ is the number of CNN layers, $D$ is the spatial dimension, $K$ is the convolution kernel size, $H$ is the count of attention heads, $N$ is the sequence length, and $d$ is the feature dimension. The AFpM activation uses *O*(*n*) for *n* neurons. Considering the model parameters and activation functions in the convolution and Transformer components, the spatial complexity is $O(N \cdot d^2 + L \cdot D^2 + C)$. The AFpM parameter is one scalar per activation layer.

**Algorithm 1:**
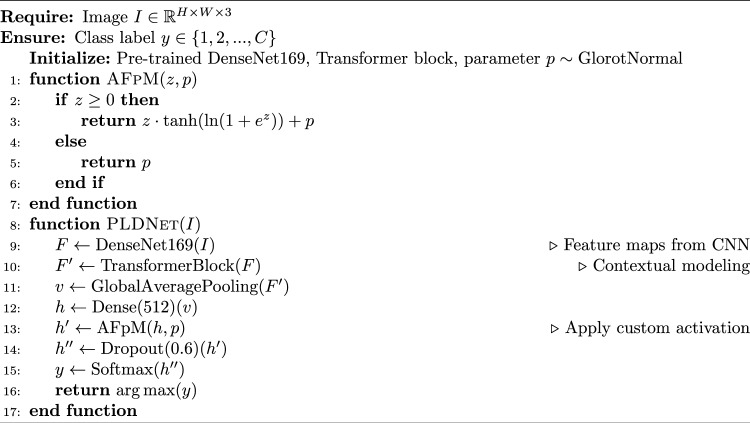
PLDNet with Adaptive Flatten p-Mish (AFpM) Activation

#### Optimization objective: constraint programming formulation

The model parameter is $\theta$ and the AFpM parameter is *p*. The classification loss (cross entropy) is minimized on the training set.

4 $$\begin{aligned} \min _{\theta , p} \quad&\mathcal {L} = -\sum _{i=1}^{N} \sum _{j=1}^{C} y_{ij} \log (\hat{y}_{ij}) \end{aligned}$$

5 $$\begin{aligned} \text {subject to} \quad&p \sim \mathcal {N}(0, \sigma ^2) \text { (initialized via GlorotNormal)} \end{aligned}$$

6 $$\begin{aligned}&\theta , p \in \mathbb {R}^{n}, \quad n \le n_{\max } \text { (parameter constraint)} \end{aligned}$$

7 $$\begin{aligned}&\text {AFpM } \in \mathcal {C}^1 \text { (differentiable activation for backpropagation)} \end{aligned}$$

#### Computational complexity classification

General image classification problems belong to the P class because the complexity of CNN inference is polynomial with respect to the input size and the number of parameters. However, optimization of neural network parameters (especially for non-convex loss graphs and learnable activation functions such as AFpM) can achieve training convergence, including:

- NP-hard subproblems: Optimization of deep networks with non-convex constraints (activation learning) is known to be NP-hard in the worst case.
- Gradient descent heuristic allows practical convergence.
- Minimizing overfitting and regularization constraints are computationally intensive (e.g. Dropout tuning = NP-hard combinatorial optimization).

While PLDNet is capable of performing polynomial inference, it requires solving an NP-hard training subproblem due to learnable parameters and deep architecture optimization. This reflects prediction is efficient, but training dynamics are difficult to optimize.

### Experimental setup

In this study, we describe the experimental configuration adopted for the training and evaluation of the proposed approach. The available data were divided into training and testing subsets using an 80:20 stratified split. We have trained our models on the PlantVillage and Mendeley datasets separately, using identical training configurations, to assess their controlled and real-world performance independently. Model optimization was carried out using the Adam optimizer with a learning rate $(\alpha )$ of 0.0001, a categorical cross-entropy loss function, a batch size of 32, and a total of 100 training epochs. To enhance convergence efficiency and mitigate performance stagnation during training, the *ReduceLROnPlateau* strategy^57^ was applied, which adaptively decreases $\alpha$ by a factor of 0.2 when the validation loss remains unchanged for three successive epochs. To minimize overfitting and encourage improved generalization, the *Early Stopping* mechanism^57^ was incorporated with a patience threshold of 15 and a minimum delta of 0.001, terminating training when the validation loss ceased to improve. The *ModelCheckpoint* callback was further employed to preserve the model weights corresponding to the minimum validation loss attained. Model development was performed using the TensorFlow framework, while Seaborn and Matplotlib were used for visualization, and Scikit-learn along with NumPy were utilized for data preprocessing within the Google Colaboratory environment.

### Performance metrics

We evaluated our proposed methodology using a number of metrics^58–60^,, including confusion matrix, precision, specificity, sensitivity, F1-score, Matthews’ coefficient (MCC), overall accuracy, and balanced accuracy^58^

Precision^58^ measures the proportion of true positive predictions among all positive predictions.

4 $$\begin{aligned} \text {Precision} = \frac{\text {TP}}{\text {TP} + \text {FP}} \end{aligned}$$

Sensitivity (Recall)^58^ measures the proportion of true positive predictions among all actual positives.

5 $$\begin{aligned} \text {Sensitivity} = \frac{\text {TP}}{\text {TP} + \text {FN}} \end{aligned}$$

Specificity^58^ measures the proportion of true negative predictions among all actual negative instances:

6 $$\begin{aligned} \text {Specificity} = \frac{\text {TN}}{\text {TN} + \text {FP}} \end{aligned}$$

F1-score^58^ is the harmonic mean of Precision and Recall, providing a balanced measure between the two.

7 $$\begin{aligned} \text {F1-score} = 2 \times \frac{\text {Precision} \times \text {Recall}}{\text {Precision} + \text {Recall}} \end{aligned}$$

Overall accuracy^58^ measures the proportion of correct predictions among all predictions.

8 $$\begin{aligned} \text {Accuracy} = \frac{\text {TP} + \text {TN}}{\text {TP} + \text {TN} + \text {FP} + \text {FN}} \end{aligned}$$

MCC^58^ is a balanced measure that takes into account all four metrics: TP, TN, FP, and FN.

9 $$\begin{aligned} \text {MCC} = \frac{\text {TP} \times \text {TN} - \text {FP} \times \text {FN}}{\sqrt{(\text {TP} + \text {FP})(\text {TP} + \text {FN})(\text {TN} + \text {FP})(\text {TN} + \text {FN})}} \end{aligned}$$

Although accuracy and F1-score^58^ are useful metrics, they may not always provide a complete picture of a classification model’s performance, especially when there is a class disparity. MCC^58^ addresses these limitations by offering a balanced measure that considers both positive and negative classes, making it a more reliable metric for evaluating classification models. Therefore, MCC^58^ is often preferred over accuracy and F1-score^58^ for its robustness and balanced nature in assessing classification performance. Moreover, we have used balanced accuracy^59^ for evaluating performance of classification. It can be defined as the average of sensitivity scores calculated for each class, making sure each class has the same effect on the overall accuracy. It plays a vital role to ensure the reliability and generalizability of classification models, particularly in scenarios with imbalanced class distributions.

10 $$\begin{aligned} \text {Balanced Accuracy} = \frac{1}{N} \sum _{i=1}^{N} \frac{TP_i}{P_i} \end{aligned}$$

Where:

$$\begin{aligned} N&: \text {Number of classes} \\ TP_i&: \text {True Positives for class } i \\ P_i&: \text {Total number of positives for class } i \end{aligned}$$

## Results and discussion

In this section, we have discussed our experimental findings in detail. First, we have presented the classification performance of our proposed model, PLDNet, with the newly developed AFpM activation function on both potato leaf disease classification datasets. Then, we have shown the effect of the data augmentation technique on the classification performance of PLDNet. Subsequently, a comparative performance analysis of AFpM with several existing activation functions^41,48–52^ has been conducted using PLDNet on both datasets to evaluate the effectiveness of AFpM. Furthermore, we have compared the classification performance of PLDNet with various pre-existing deep CNN models, like DenseNet201^44^, DenseNet169^44^, InceptionV3^45^, ResNet50^46^, VGG16^46^, and MobileNetV2^47^, and also presented a comparative performance analysis between our proposed methodology and the previous works.

### Classification performance of PLDNet with AFpM

**Fig. 5:**
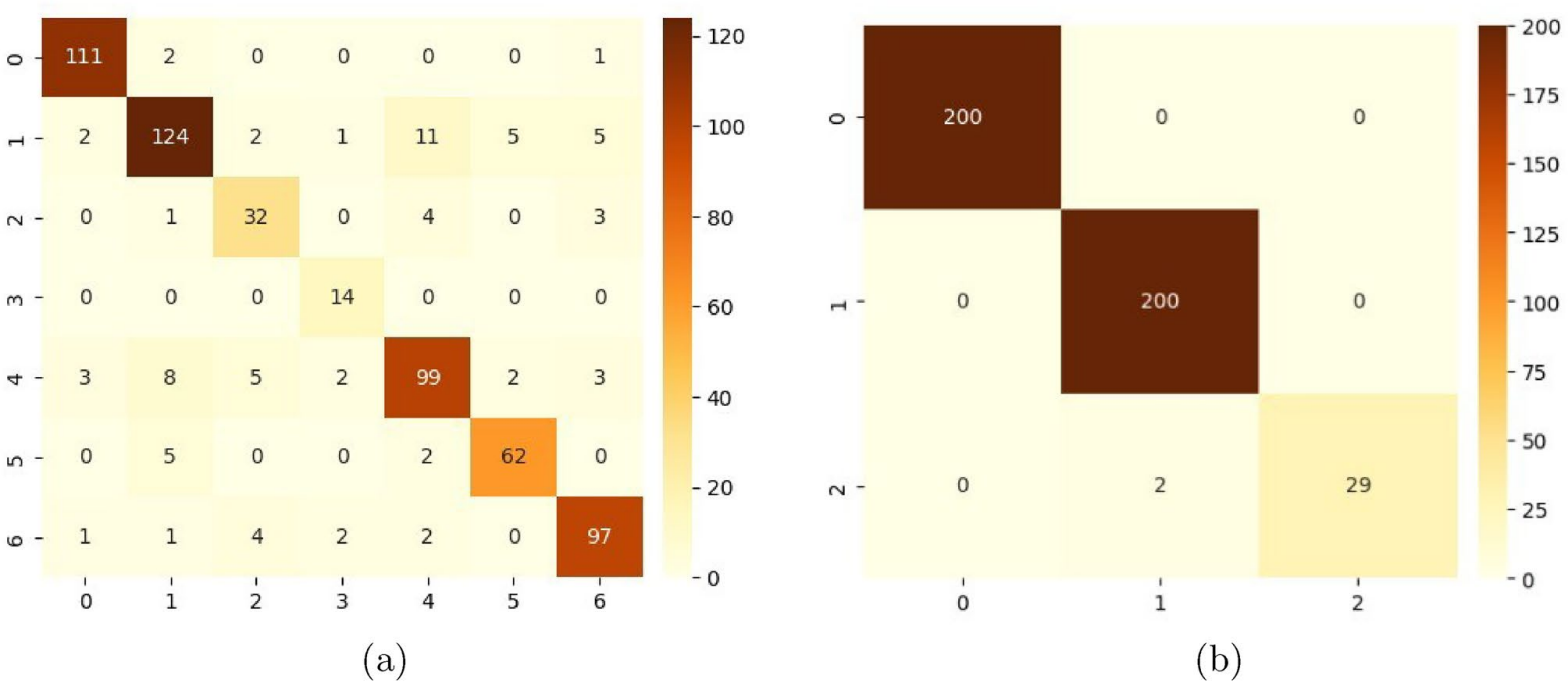
Confusion matrix obtained by PLDNet model on: (**a**) Mendeley dataset; (**b**) PlantVillage dataset.

**Fig. 6:**
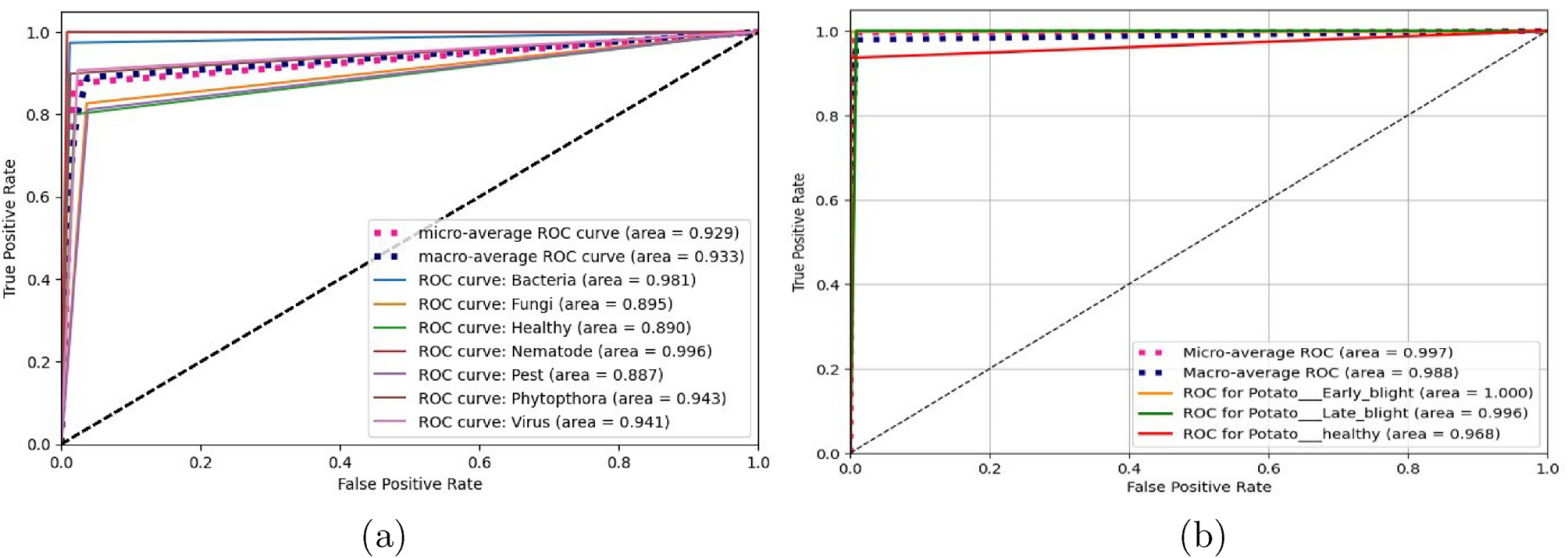
ROC curve obtained by PLDNet model on: (**a**) Mendeley dataset; (**b**) PlantVillage dataset.

We have applied our proposed PLDNet model along with the activation function AFpM and validated our methodology using two different potato leaf image classification datasets: a new dataset available on Mendeley^28^ and the PlantVillage dataset^40^. Table 4 contains different parameters such as precision, specificity, sensitivity, F1-score, MCC, overall accuracy, and balanced accuracy obtained by the PLDNet model for both potato leaf datasets. PLDNet has achieved approximately 87.50% overall classification accuracy on the Mendeley dataset, and the same for the PlantVillage dataset is 99.54%. Figure 5 depicts the confusion matrix obtained by the proposed PLDNet model for both datasets. It can be observed from Figure 5(a) that PLDNet has correctly classified all images belonging to Nematode, and the least class-wise accuracy has been acquired in the Healthy class. Similarly, from Figure 5(b), we can see that PLDNet accurately classified all images except two healthy leaf images for the PlantVillage dataset. Next, we have shown the receiver operating characteristic (ROC) curves along with the area under the curve (AUC) values for each class for both datasets in Figure 6. For the Mendeley dataset, the class-wise AUC values achieved by PLDNet are 0.981 for Bacteria, 0.895 for Fungi, 0.890 for Healthy, 0.996 for Nematode, 0.887 for Pest, 0.943 for Phytopthora, and 0.941 for Virus. On the other hand, for the PlantVillage dataset, the class-wise AUC values attained by PLDNet are 1.00 for Early blight, 0.996 for Late blight, and 0.968 for healthy.

**Table 4:** Classification performance achieved by PLDNet on both potato leaf disease datasets.

Dataset	Classes	Precision (%)	Specificity (%)	Sensitivity (%)	F1-score (%)	MCC (%)	Overall accuracy (%)	Balanced accuracy (%)
Mendeley^28^	Bacteria	94.87	98.80	97.37	96.10	84.83	87.50	88.81
	Fungi	87.94	96.35	82.87	85.22			
	Healthy	74.42	98.09	80.00	77.11			
	Nematode	73.68	99.17	100.00	84.85			
	Pest	83.90	96.15	81.15	82.50			
	Phytopthora	89.86	98.72	90.65	89.81			
	Virus	88.99	97.64	90.65	89.81			
PlantVillage^40^	Potato_Early_blight	100.00	100.00	100.00	100.00	99.32	99.54	97.85
	Potato_Late_blight	99.01	99.13	100.00	99.50			
	Potato_healthy	100.00	100.00	93.55	96.67			

### Effect of data augmentation

In this section, we have shown the effect of data augmentation on potato leaf disease classification. Table 5 depicts the comparative classification performance of the PLDNet model on both Mendeley and PlantVillage before and after applying the data augmentation technique to the training data. Using the data augmentation technique, the PLDNet model has achieved approximately 87.50% overall accuracy on the Meneley dataset and 99.54% overall accuracy on the PlantVillage dataset. Without any data augmentation technique, PLDNet achieved nearly 86.04% and 96.55% overall accuracy on Mendeley and PlantVillage datasets, respectively.

The observed performance gains are due to the model’s greater exposure to a variety of leaf images, including variations in orientation, scale, and texture. These augmentations improve the model’s generalization ability by emulating real-world biases and reducing overfitting, especially for underrepresented classes. This benefit is particularly evident in the PlantVillage dataset, where data augmentation helps balance learning due to the small number of samples in the healthy class. Similar results are observed in the more complex and noisier Mendeley dataset, where data augmentation helps better feature learning and class separation, despite differences in image background and lighting. Such results highlight the necessity of data augmentation as a key step in training deep learning models for robust agricultural applications.

**Table 5:** Effect of data augmentation on classification performance of PLDNet across both datasets.

Data augmentation	Dataset	Average precision (%)	Average specificity (%)	Average sensitivity (%)	Average F1-score (%)	Average MCC (%)	Overall accuracy (%)	Balanced accuracy (%)
Yes	Mendeley	87.62	97.43	87.50	87.48	84.83	87.50	88.81
	PlantVillage	99.54	99.60	99.54	99.53	99.32	99.54	97.85
No	Mendeley	86.13	97.11	86.04	85.97	83.06	86.04	84.35
	PlantVillage	96.41	97.90	96.52	96.46	94.64	96.52	97.21

### Comparison of AFpM with different activation functions

We have compared the classification performance of AFpM with six different pre-existing activation functions, including ReLU^41^, Leaky ReLU^48^, GELU^49^, Swish^50^, Mish^51^, and PFpM^52^, in the PLDNet model. Table 6 presents the classification performance of PLDNet on both potato leaf disease datasets using different activation functions. It can be observed that PLDNet with AFpM has attained the highest overall classification accuracy of 87.50% on the Mendeley dataset and 99.54% overall accuracy on the PlantVillage dataset. Among the pre-existing activation functions, PLDNet has achieved 85.06% and 97.68% overall accuracy on Mendeley and PlantVillage datasets, respectively, using the PFpM activation function. In contrast, the PLDNet model with the ReLU activation function has acquired an overall accuracy of only 83.12% and 92.81% for the same datasets. Therefore, the PLDNet model has achieved promising classification accuracy on both potato leaf disease datasets using different activation functions, especially AFpM.

**Table 6:** Effect of different activation functions on the classification performance of PLDNet across both datasets.

Activation function	Dataset	Average precision (%)	Average specificity (%)	Average sensitivity (%)	Average F1-score (%)	Average MCC (%)	Overall accuracy (%)	Balanced accuracy (%)
ReLU	Mendeley	83.13	96.21	83.12	83.12	79.40	83.12	78.91
	PlantVillage	92.74	95.60	92.81	92.78	88.46	92.81	94.20
Leaky ReLU	Mendeley	83.33	96.25	83.28	83.30	79.60	83.28	79.93
	PlantVillage	93.04	95.80	93.04	93.04	88.84	93.04	94.42
GELU	Mendeley	83.64	96.32	83.60	83.62	79.99	83.60	80.24
	PlantVillage	94.16	96.26	94.20	94.18	90.52	94.20	95.23
Swish	Mendeley	83.96	96.37	83.93	83.95	80.37	83.93	81.39
	PlantVillage	94.43	96.46	94.43	94.43	90.89	94.43	95.45
Mish	Mendeley	84.27	96.47	84.25	84.26	80.77	84.25	81.62
	PlantVillage	95.32	97.26	95.36	95.34	92.71	95.36	96.31
PFpM	Mendeley	85.10	96.62	85.06	85.08	81.78	85.06	82.16
	PlantVillage	97.61	98.36	97.68	97.64	96.55	97.68	98.02
AFpM	Mendeley	**87.62**	**97.43**	**87.50**	**87.48**	**84.83**	**87.50**	**88.81**
	PlantVillage	**99.54**	**99.60**	**99.54**	**99.53**	**99.32**	**99.54**	**97.85**

### Comparison of PLDNet with different state-of-the-art CNN models

In Table 7, we have presented a comparative classification performance between PLDNet and a few state-of-the-art deep CNN models like DenseNet201^44^, DenseNet169^44^, InceptionV3^45^, ResNet50^46^, VGG16^46^, and MobileNetV2^47^ on both Mendeley and PlantVillage datasets. PLDNet surpassed its counterparts in terms of different performance metrics, such as average precision, specificity, sensitivity, F1-score, MCC, overall accuracy and balanced accuracy on both datasets. PLDNet has attained the highest overall classification accuracy of 87.50% on the Mendeley dataset and 99.54% overall accuracy on the PlantVillage dataset. Similarly, among the existing state-of-the-art CNN models, DenseNet169 has achieved the highest overall accuracy of 82.47% and 91.88% on the Mendeley and the PlantVillage datasets, respectively.

**Table 7:** Comparison of different CNN models on classification performance across both datasets.

Model	Dataset	Average precision (%)	Average specificity (%)	Average sensitivity (%)	Average F1-score (%)	Average MCC (%)	Overall accuracy (%)	Balanced accuracy (%)
DenseNet201	Mendeley	82.75	96.92	82.31	82.28	78.42	82.31	77.99
	PlantVillage	91.62	94.60	91.65	91.63	86.33	91.65	93.12
DenseNet169	Mendeley	82.67	96.09	82.47	82.42	78.66	82.47	80.32
	PlantVillage	91.80	94.62	91.88	91.84	86.63	91.88	93.25
InceptionV3	Mendeley	79.72	95.13	79.22	79.11	74.79	79.22	75.61
	PlantVillage	91.62	94.60	91.65	91.63	86.33	91.65	93.12
ResNet50	Mendeley	82.27	95.95	82.14	82.18	78.22	82.14	81.93
	PlantVillage	90.54	93.59	90.49	90.51	84.23	90.49	92.04
VGG16	Mendeley	76.32	94.42	75.97	75.29	70.86	75.97	73.30
	PlantVillage	90.20	93.56	90.02	90.11	83.61	90.02	91.79
MobileNetV2	Mendeley	78.82	95.28	78.41	78.33	73.86	78.41	75.14
	PlantVillage	89.16	93.10	88.86	89.01	81.96	88.86	90.98
PLDNet	Mendeley	**87.62**	**97.43**	**87.50**	**87.48**	**84.83**	**87.50**	**88.81**
	PlantVillage	**99.54**	**99.60**	**99.54**	**99.53**	**99.32**	**99.54**	**97.85**

### Contextualization of PlantVillage vs. Mendeley results

The very high accuracy achieved on PlantVillage (99.54%) should be interpreted in light of the dataset characteristics. PlantVillage images are largely studio-quality, captured under controlled illumination with clean, homogeneous backgrounds and limited acquisition noise; therefore, near-ceiling performance is commonly reported on this benchmark and does not necessarily reflect real-world robustness. In contrast, the Mendeley dataset contains field images with substantial domain shift, including background clutter, lighting variability (shadows and over/under-exposure), viewpoint changes, partial occlusions, and sensor/compression artifacts. The reduced accuracy on Mendeley (87.50%) thus primarily reflects this increased difficulty rather than a failure of the proposed approach.

Despite these challenges, PLDNet remains comparatively robust on Mendeley due to three design choices. First, the applied data augmentation (Table 5) exposes the model to synthetic variations in orientation, scale, and appearance that partially emulate field conditions. Second, the hybrid CNN–Transformer structure supports both local texture learning and global contextual reasoning, which helps suppress irrelevant background patterns and improves class separation under clutter. Third, the adaptive AFpM activation improves nonlinear feature shaping and gradient flow, enabling more discriminative representations in noisy settings, as reflected by consistent gains over ReLU, Mish, Swish, and PFpM (Table 6). Overall, the PlantVillage–Mendeley gap highlights the importance of evaluating on real-world imagery; accordingly, we emphasize that the practical contribution of PLDNet lies in its improved generalization on challenging field data while maintaining strong performance on curated benchmarks.

### Ablation study

An ablation study has been conducted to systematically evaluate the contribution of each architectural component in the proposed PLDNet framework. Starting from the DenseNet169 backbone as the baseline model, successive components have been incrementally added, including a Transformer module and different activation functions, to assess their individual and combined impact on classification performance. Experiments have been conducted on two benchmark datasets, Mendeley and PlantVillage, using the same training and evaluation protocol to ensure a fair comparison. The baseline DenseNet169 model provides a strong initial performance on both datasets; however, incorporating the Transformer module with a standard ReLU activation yields only marginal improvements, indicating limited gains from contextual modeling when paired with conventional activations. Replacing ReLU with the Mish activation leads to a more noticeable performance increase across most used metrics, demonstrating improved feature smoothness and gradient flow.

The best performance is achieved by integrating the proposed AFpM activation function within the DenseNet169 + Transformer architecture, forming the complete PLDNet model. As shown in Table 8, PLDNet consistently outperforms all other configurations on both datasets across all evaluation metrics, including precision, specificity, sensitivity, F1-score, Matthews Correlation Coefficient (MCC), overall accuracy, and balanced accuracy. Notably, PLDNet achieves near-perfect performance on the PlantVillage dataset, highlighting the effectiveness of the proposed architectural design and activation strategy. These results confirm that each component contributes positively to the final performance, with the combination of the Transformer module and the AFpM activation function playing a critical role in enhancing discriminative capability and robustness.

**Table 8:** Ablation study results with different model configurations across Mendeley and PlantVillage datasets.

Model configuration	Dataset	Avg. precision (%)	Avg. specificity (%)	Avg. sensitivity (%)	Avg. F1-score (%)	Avg. MCC (%)	Overall accuracy (%)	Balanced accuracy (%)
DenseNet169 (Baseline)	Mendeley	82.67	96.09	82.47	82.42	78.66	82.47	80.32
	PlantVillage	91.80	94.62	91.88	91.84	86.63	91.88	93.25
DenseNet169+ Transformer + ReLU	Mendeley	83.13	96.21	83.12	83.12	79.40	83.12	78.91
	PlantVillage	92.74	95.60	92.81	92.78	88.46	92.81	94.20
DenseNet169+ Transformer + Mish	Mendeley	84.27	96.47	84.25	84.26	80.77	84.25	81.62
	PlantVillage	95.32	97.26	95.36	95.34	92.71	95.36	96.31
DenseNet169+ Transformer + AFpM **(PLDNet)**	Mendeley	**87.62**	**97.43**	**87.50**	**87.48**	**84.83**	**87.50**	**88.81**
	PlantVillage	**99.54**	**99.60**	**99.54**	**99.53**	**99.32**	**99.54**	**97.85**

### Comparison with existing models

To evaluate the effectiveness of the proposed PLDNet model, we conducted a comparative performance analysis between PLDNet and the literature for potato leaf disease classification. Table 9 presents a summary of existing models, specifying the datasets employed, model architectures, and their respective classification accuracies. Notably, the majority of prior studies focused solely on the PlantVillage dataset-an ideal, controlled dataset with minimal noise. Although some methods, such as those by Tambe et al. (2023)^19^ and Lanjewar et al. (2024)^27^, achieved high accuracy values of 99.18% and 99.00%, respectively, they were assessed only on this curated dataset. In contrast, PLDNet demonstrates robust performance across both PlantVillage and Mendeley datasets, attaining 99.54% accuracy on PlantVillage and 87.50% on the more challenging Mendeley dataset, which contains real-world images with complex backgrounds, lighting variations, and noise. Furthermore, models like EfficientNetV2B3^28^ and RCA-Net^31^ achieved lower performance on Mendeley, reporting 73.63% and 78.14%, respectively. It highlights PLDNet’s superior generalization to diverse field conditions. The improved classification accuracy of PLDNet can be attributed to its hybrid CNN-Transformer architecture and the introduction of the Adaptive Flexible parametric Mish (AFpM) activation function, which learns optimal nonlinearities during training. This data-driven activation mechanism contributes significantly to the model’s adaptability and classification accuracy under real-world variations.

**Table 9:** Comparative performance between the proposed model and the literature on PlantVillage and Mendeley datasets.

**Author (Year)**	**Dataset**	**Applied model**	**Accuracy (%)**
Islam et al. (2017)^13^	PlantVillage	SVM	95.00
Iqbal et al. (2020)^14^	PlantVillage	Global Feature + Random Forest	97.00
Tiwari et al. (2020)^15^	PlantVillage	VGG19 + Logistic Regression	97.80
Kumar et al. (2021)^16^	PlantVillage	FFNN	96.50
Ali et al. (2022)^17^	PlantVillage	Enhanced EfficientNet	97.22
Patil et al. (2023)^18^	PlantVillage	CNN	98.00
Tambe et al. (2023)^19^	PlantVillage	CNN	99.18
Muthuraja et al. (2023)^20^	PlantVillage	ResNet152	98.78
Arshad et al. (2023)^21^	PlantVillage	Ensemble (VGG19 + Inception-V3)	98.66
Kumar et al. (2023)^22^	PlantVillage	CNN	98.83
Mathur et al. (2024)^23^	PlantVillage	VGG19	93.00
Roy et al. (2024)^24^	PlantVillage	CNN	97.16
Khan et al. (2024)^25^	PlantVillage	CNN	97.33
Paul et al. (2024)^26^	PlantVillage	ResNet-50	98.79
Lanjewar et al. (2024)^27^	PlantVillage	Modified DenseNet	99.00
Mhala et al. (2025)^30^	Mendeley	DenseNet201	81.31
Park et al. (2025)^31^	Mendeley	RCA-Net	78.14
Shabrina et al. (2023)^28^	PlantVillage	EfficientNetV2B3	98.15
	Mendeley	EfficientNetV2B3	73.63
Sinamenye et al. (2025)^29^	PlantVillage	EfficientNetV2B3 + ViT	98.15
	Mendeley	EfficientNetV2B3 + ViT	85.06
**Proposed Method**	PlantVillage	**PLDNet (CNN + Transformer + AFpM)**	**99.54**
	Mendeley	**PLDNet (CNN + Transformer + AFpM)**	**87.50**

### Statistical analysis

**Fig. 7:**
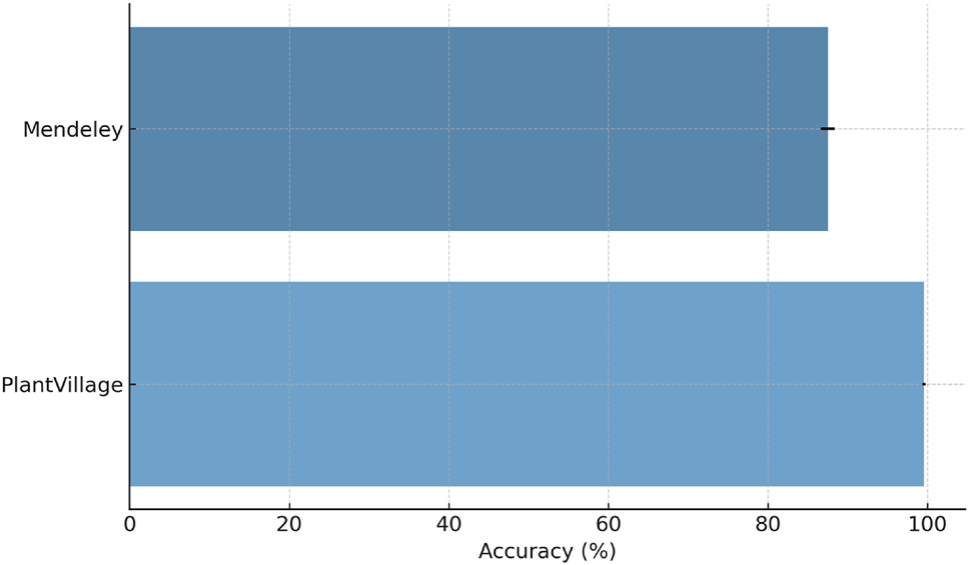
Statistical Analysis of PLDNet Performance.

To robustly evaluate the AFpM-based PLDNet model, we calculated statistical metrics across five independent experiments with random training and test set splits. On the PlantVillage dataset, PLDNet achieved an average accuracy of 99.54% with a standard deviation of 0.18%, demonstrating high consistency across all experiments. Similar results were obtained on the Mendeley dataset: the model achieved an average accuracy of 87.50% with a standard deviation of 0.47%. The small standard deviations in both datasets demonstrate the model’s stability and reliability across different training conditions. In addition to standard deviations, 90% confidence intervals were calculated to ensure statistical performance. For the PlantVillage dataset, the confidence intervals for overall accuracy ranged from 99.36% to 99.72%, while for the Mendeley dataset, the confidence intervals ranged from 86.93% to 88.08%. These intervals confirm that the observed performance gains are not due to random initialization or chance, but rather to the underlying architecture and activation function. The statistical consistency of metrics such as precision, recall, and F1 score highlights the reliability of PLDNet and its potential for practical application in potato disease diagnosis (see Fig. 7).

### Limitations and future work

Although the proposed PLDNet framework demonstrates strong performance on both a controlled benchmark dataset (PlantVillage) and a real-world dataset (Mendeley), certain limitations remain and point to important directions for future research. First, the current evaluation is limited to two publicly available datasets. While these datasets capture complementary characteristics-studio-quality images versus field-acquired images-they do not fully encompass the diversity of real-world agricultural conditions. In particular, no cross-dataset generalization experiment (e.g., training on PlantVillage and testing on Mendeley) was conducted in this study, as such a setting would introduce severe domain mismatch and label distribution shifts. Consequently, although PLDNet exhibits improved robustness on the more challenging Mendeley dataset compared to existing methods, its generalization across unseen domains cannot yet be fully guaranteed.

Second, despite the use of extensive data augmentation to simulate variations in orientation, scale, and illumination, PLDNet still experiences performance degradation under extreme field conditions, such as heavy occlusion, overlapping leaves, severe background clutter, and poor or uneven lighting. These challenges highlight the inherent difficulty of deploying vision-based disease classification models in unconstrained agricultural environments. Additionally, the proposed AFpM activation function, while enhancing adaptability through learnable nonlinearities, increases computational complexity and may raise the risk of overfitting when training data are scarce. Similarly, the inclusion of a Transformer module improves contextual modeling but introduces additional computational overhead, which may limit real-time deployment on resource-constrained or embedded devices.

Future work will therefore focus on several complementary directions to strengthen generalization claims. First, we plan to perform explicit cross-dataset and cross-domain evaluations, including training on curated datasets and testing on field images, to better quantify domain shift effects. Second, domain adaptation and domain generalization techniques, such as feature alignment, adversarial learning, or self-supervised pretraining on unlabeled field data, will be explored to improve robustness under real-world conditions. Third, model optimization strategies, including pruning, quantization, and lightweight attention mechanisms, will be investigated to reduce computational cost and enable deployment on edge devices. Finally, expanding the dataset to include images from multiple geographical regions, growth stages, and environmental conditions, together with the integration of explainable AI methods (e.g., Grad-CAM or attention visualization), will further enhance model interpretability, reliability, and practical applicability in real agricultural settings.

## Conclusion

This study presents a novel deep learning framework, termed PLDNet, which integrates Convolutional Neural Network (CNN) and Transformer components along with a newly designed activation function, AFpM, to achieve precise classification of potato leaf diseases. The proposed PLDNet model has achieved 99.54% accuracy on PlantVillage dataset and 87.50% on Mendeley dataset, demonstrating clear improvements over existing CNN, hybrid, and activation-based approaches, particularly under complex real-world imaging conditions. The AFpM activation function enhances the network’s adaptability by enabling dynamic nonlinear transformations, which contribute to improved classification performance. Despite its relatively higher computational cost resulting from the inclusion of the Transformer layer and trainable activation parameters, PLDNet establishes a strong benchmark in terms of generalization and robustness. Future research directions include refining the model architecture for edge-level deployment, incorporating explainable artificial intelligence techniques, and investigating self-supervised learning strategies to minimize dependence on annotated agricultural datasets.

## Data Availability

The dataset used in this study is available at: https://data.mendeley.com/datasets/ptz377bwb8/1 and https://www.kaggle.com/datasets/emmarex/plantdisease.

## Code availability

The GitHub codebase will be made public once the paper is accepted.
